# Wavelet-Based Kalman Smoothing Method for Uncertain Parameters Processing: Applications in Oil Well-Testing Data Denoising and Prediction

**DOI:** 10.3390/s20164541

**Published:** 2020-08-13

**Authors:** Xin Feng, Qiang Feng, Shaohui Li, Xingwei Hou, Mengqiu Zhang, Shugui Liu

**Affiliations:** 1 State Key Laboratory of Precision Measuring Technology and Instruments, Tianjin University, Tianjin 300072, China; fengxin182@tju.edu.cn (X.F.); houxingwei@tju.edu.cn (X.H.); mqzhangvip@tju.edu.cn (M.Z.); 2 CNPC Bohai Drilling Engineering Company Ltd., Tianjin 300457, China; feng_qiang@cnpc.com.cn; 3 Tianjin Research Institute of Water Transport Engineering, Tianjin 300000, China; hanhongsheng@tiwte.ac.cn

**Keywords:** low-distortion processing, oil well-testing data, wavelet analysis, Kalman prediction, data smoothing, data compression, robustness

## Abstract

The low-distortion processing of well-testing geological parameters is a key way to provide decision-making support for oil and gas field development. However, the classical processing methods face many problems, such as the stochastic nature of the data, the randomness of initial parameters, poor denoising ability, and the lack of data compression and prediction mechanisms. These problems result in poor real-time predictability of oil operation status and difficulty in offline interpreting the played back data. Given these, we propose a wavelet-based Kalman smoothing method for processing uncertain oil well-testing data. First, we use correlation and reconstruction errors as analysis indicators and determine the optimal combination of decomposition scale and vanishing moments suitable for wavelet analysis of oil data. Second, we build a ground pressure measuring platform and use the pressure gauge equipped with the optimal combination parameters to complete the downhole online wavelet decomposition, filtering, Kalman prediction, and data storage. After the storage data are played back, the optimal Kalman parameters obtained by particle swarm optimization are used to complete the data smoothing for each sample. The experiments compare the signal-to-noise ratio and the root mean square error before and after using different classical processing models. In addition, robustness analysis is added. The proposed method, on the one hand, has the features of decorrelation and compressing data, which provide technical support for real-time uploading of downhole data; on the other hand, it can perform minimal variance unbiased estimates of the data, filter out the interference and noise, reduce the reconstruction error, and make the data have a high resolution and strong robustness.

## 1. Introduction

In the process of petroleum exploration, the downhole pressure–time relationship in a test well can be obtained through well opening and closing operations. A reasonable real-time data processing method is the prerequisite for successfully explaining the formation structure and oil and gas distribution [1]. However, due to the interference of mechanical vibration, electrical faults, and uncertain noise, useful formation signals are often submerged in a large number of fluctuations and burrs, which are shown as non-stationary and time-varying [2]. These disturbances will not only affect the real-time prediction of downhole operation status, but also lead to inaccuracy of offline playback data, resulting in the inconsistency between the pressure measured from the surface and that from downhole [3], the misjudgment of the interpretation model mode [4], and even major accidents [5]. Logging the wrong production parameter will also increase the uncertainty of reservoir simulation and will result in the mistake of making a decision. In addition, the development of large-bandwidth transmission technology has made it easier and more convenient to routinely collect large underground data. However, at the same time, it also brings huge challenges for engineers to the management, filtering, reduction, and interpretation of these large data sets. Kikani [6] emphasized the importance of data denoising, data reduction, and trend analysis as early as 1998. He introduced the wavelet transform to accomplish the time–frequency analysis of long-term pressure transient data. For many years, although large works have been done to address individual issues, none of them have been systematically studied until now.

Therefore, to meet the needs of downhole data compression transmission and interpretation, how to efficiently extract the change trend containing well-testing operation characteristics from the strong noise background with variable frequency and compress the data have become important focuses of studying downhole parameter processing methods [7]. Given this, this paper establishes the following three purposes:

1. Filter out the noise or interference signals on the premise of ensuring undistorted original well-testing information.

2. Add the data prediction function in the data denoising process to meet the requirements of data noise reduction and underground accident early warning in actual production simultaneously.

3. Find a proper mathematical expression suitable for reversible data compression, convenient data storage, and upload, providing the possibility for transmitting the downhole data to the ground accurately and in real-time.

To filter out noises, enhance useful information, and obtain the dynamic change course of all kinds of geological parameters of oil–gas wells, the first and foremost task is denoising method selection. Classical data denoising methods mostly adopt simple moving average (SMA) [8] and frequency domain filtering [9]. However, the direct use of the SMA method will cause a loss of useful data information, resulting in a decrease in the recognizability of the data [10]. As a classic frequency domain filtering method, Fourier can filter out high frequency interference, but it cannot perform local analysis [11]. Compared with the above methods, the wavelet multiscale transform has good time–frequency domain resolution capabilities that make it extensively applied in reservoir description, noise removal, and data analysis [12], from the earliest seismic signal processing [13] to transient pressure data denoising [14] to parameter estimation [15].

As for the application of wavelet transform in oil and gas well exploration, in recent years, the method has been widely used in logging while drilling (LWD) data processing [16], downhole data boundary determination [17], and historical data reconstruction [18]. Wang [3] used the wavelet transform method to analyze the downhole transient pressure data and proved that the wavelet method can be applied to remove data noise. Athichanagorn [19] developed a wavelet-based algorithm for most of the data processing steps, such as outlier removal, denoising, transient identification, and data reduction by using the long-term data from permanent gauges. Based on Knutson Towboat Company (KTB) logging data, Amrita [20] conducted continuous wavelet transform (CWT) analysis using different decomposition scales. However, due to the difference in time–frequency characteristics of different wavelet bases, different wavelet bases often output different results when processing the same signal. To achieve the detection of a stress concentration zone for oil well casing, Liu [21] used the median filter method and wavelet transform to reduce the noises of the signal. The median filter replaces the center signal value with the signal value from the middle of the sorted list, which will lose a lot of useful information, causing a huge deviation between the filtered signal and the original signal. Zheng [22] presented a wavelet transform-based approach for recovering the production history from the measured permanent downhole gauge (PDG) pressure. By assuming that the reservoir system is linear, they realized the production evaluation of the reservoir. However, the fact is that, for a reservoir with variable properties, the above assumption is not valid. Later, Wang [23] used the Haar wavelet transform for analyzing non-stationary PDG pressure signal, to achieve the purpose of identifying the changes in reservoir properties. Although the above studies deal with issues associated with the processing of pressure data using wavelet techniques, they only touch on a single or a couple of problems related with oil well data. Research on well testing data is even rarer. These works just use different algorithms to process the data in turn, and do not analyze the influence of different wavelet configuration parameters on the processing results. In addition, the existing processing methods only filter the data and lack data prediction mechanisms, so they cannot meet the urgent requirements of special production occasions such as downhole decision-making or early warning.

**Figure 1:**
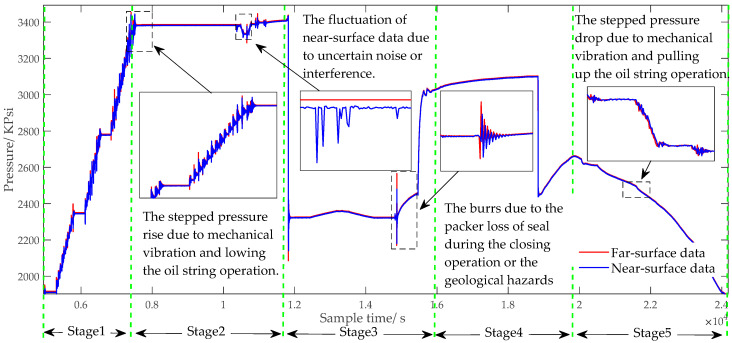
Diagram of oil well-testing data characteristics analysis. Five stages in the complete well-testing process are: Stage1: Lowering the oil string. Stage 2: Waiting stage. Stage 3: Well opening. Stage 4: Well closing. Stage 5: Pulling up the oil string.

Moreover, some stage curves have similar characteristics with noise, which will make data processing more difficult. Figure 1 analyzes the characteristics of oil well-testing data. Generally, the complete well-testing operation process consists of five stage. The near-surface data are easy to transmit, but they are not as accurate as the far-surface data. In the waiting stage, because there is no downhole operation, the far-surface data remain stable and the pressure curve does not fluctuate too much. By contrast, the near-surface data in the same working stage have fluctuations due to uncertain noise or interference. These differences between near and far surface data are exactly what need to be removed in the filtering process. Meanwhile, the noises are mixed with real downhole geological pressure signals, which have similar characteristics with normal signal. Taking the stepped pressure rise as an example, even if the data characteristics are the same, the causes may be different, such as mechanical vibration, the operation of lowering the oil string, and the negligent use of oilfield workers. Similarly, there also exist burrs and stepped pressure due to the variations in downhole geological conditions and surface operation environment. If the data processing method is not appropriate, it will be very hard to identify whether the above similar characteristics is useful or not, and the useful signal will be easily filtered out as noise. Besides physical changes in the test well, the pressure gauge data acquisition system itself may pose a problem. In some cases, pressure data are stored with low precision, creating stepped change and noise [19]. All of the above situations bring much more uncertainty and ambiguity to the well-testing data processing.

The above similarities and the randomness of engineering parameters show that it is difficult to accurately describe oil and gas operations using simple mathematical formulas. It is necessary to use random system methods for non-deterministic analysis. The good news is that we found that Kalman has advantages in data smoothing and prediction, and can be used to separate dynamic noise and observation noise [24]. It is not only widely used in the fields of medical [25], aviation [26], geology [27], and disaster prediction [28], but also plays an important role in evaluating oil well reservoir parameters [29,30], model parameter correction [31,32], and reservoir dynamic monitoring [33,34]. To improve the performance of reservoir prediction, Raghu [35] used a Kalman filter to estimate the spatial permeability distribution. Xue [36] used the Kalman state-space model to correct the triaxial parameters near the bit. By using the concentration of radioactive elements, Soltani [37] proposed a Kalman-based method for solving log estimation. Mahdianfar [38] established a program-controlled hydraulic model for drilling operations and used Kalman filters for parameter estimation. However, the above researchers only considered the removal of mechanical interference and random noise, and ignored the influence of the algorithm parameters on the final processing results, resulting in poor robustness, and mistakenly removing useful information as noise. Fortunately, particle swarm optimization (PSO) is a type of full-scale random algorithm of optimization and has been widely used in petroleum exploration. The algorithm not only seeks the optimal position of individual particles but also keeps tracking the overall optimal value. To estimate reservoir properties, Adibifard [39] used PSO to implement nonlinear regression in well test analysis. Zhang [40] used PSO-based BP neural networks to predict reservoir parameters by using dynamic production information. Lang [41] studied an oil well production scheduling problem for the light load oil well during petroleum field exploitation. He proved that the PSO-based method can obtain high quality schedules within a much shorter running time. These studies show that PSO brings hope for parameter optimization.

As for the ensemble method, Kalman prediction and wavelet transform are functionally complementary. Combining the two can not only improve the resolution of the data, but also realize the dynamic prediction of engineering parameters [42]. Hong [43] proposed a Kalman Filtering method for wavelet estimation and decomposition of Random Signals. However, the method is developed based on the standard Kalman filtering and needs a set of Monte Carlo simulations. The limitation of the algorithm itself, the high consumption of the hardware, and the difficulty of algorithm transplantation bring lots of obstacles for its application. Soltani [44] designed a fuzzy Kalman filter based on a wavelet smoother to overcome the uncertainty. However, due to the complexity of the processing model and a large number of samples, the method is poorly implantable and cannot be popularized in special underground occasions that require real-time prediction and uploading.

In summary, the existing methods face great challenges in dealing with the problems coming from the occasions that urgently need multi-control parameter optimization, real-time data feedback, and prediction. Considering that the direct data prediction for signals with noises may lead to large uncertainties in parameter estimates or even a wrong prediction, we believe that aberrant sections of the data should be removed before data prediction. Therefore, to address the aforementioned challenges, a wavelet-based Kaman smoothing idea for well-testing data processing is coming up. The contributions are summarized as follows:

1. We are the first to integrate the wavelet transform method, Kalman prediction, and Kalman smoothing and use their integration for well-testing data processing.

2. An optimal wavelet parameter determination scheme for obtaining the optimal combination of decomposition scale and vanishing moment is proposed by taking the correlation and reconstruction errors as indicators.

3. A near-surface well-testing data measurement system of online Kalman prediction and offline Kalman smoothing is developed.

4. This paper takes the far-surface well-testing data as standard data, and proposes a wavelet-based Kalman smoothing method for near-surface well-testing data, which meet the requirements of data filtering, data compression, data prediction, and data smoothing.

The rest of this paper is organized as follows. Section 2 follows the process of method formulating to explain the working principles of different methods used in the proposed method. Section 3 describes the proposed wavelet-based Kalman smoothing measurement system and gives the detailed workflow and the algorithm flow. Section 4 presents the experimental simulations and results, and demonstrates the feasibility of the proposed scheme by analyzing the data reconstruction effects under different parameter configurations. Section 5 discusses the results and declares our future work. Section 6 concludes the paper.

## 2. Wavelet-Based Kalman Smoothing Method

**Figure 2:**
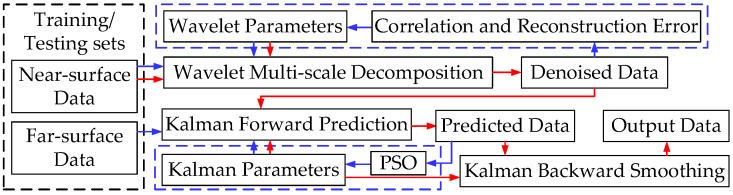
Wavelet-based Kalman smoothing (WBKS) algorithm flowchart. The blue dotted line and arrows represent the training process; the red arrows represent the testing process.

In view of weak real-time accurate data prediction and offline data analysis and interpretation capabilities due to the complex working conditions and the failure of engineering data denoising, a wavelet-based Kalman smoothing (WBKS) method to process uncertain downhole data was developed. This method combined the optimization of model parameters with data filtering and prediction, and adopted the technical route of multiscale decomposition, particle swarm optimization (PSO), Kalman prediction, and Kalman smoothing in turn. First, based on the measured downhole well-testing samples, take correlation as an index and optimize the decomposition scale and disappearance moment of the Symlets wavelet family through the iterative learning of training samples. Then, use PSO to find the optimal variance of Kalman prediction error and observation error. Finally, with the best optimization parameters, Kalman forward prediction and backward smoothing are sequentially performed on the testing samples to complete the entire processing. The flowchart of WBKS is shown in Figure 2.

### 2.1. Wavelet Transformation and Compression

**Figure 3:**
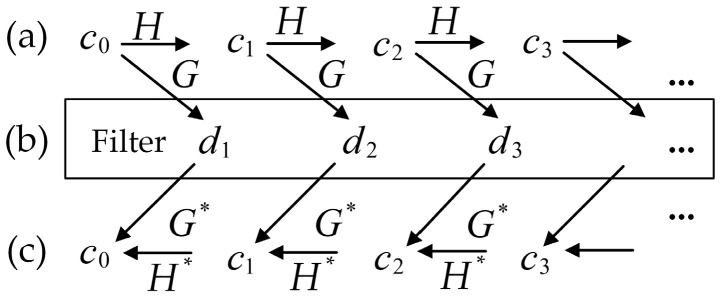
The flow of wavelet decomposition, threshold processing, and reconstruction. The data processing steps in this figure are as follows: (**a**) Decompose the well-testing data into multi-layers by Symlets wavelet. The decomposition is carried out only for low-frequency parts each time; $c_{0}$ to $c_{3}$ are the frequency details of the signal with different decomposition levels. The level is determined as needed. (**b**) Threshold process the high frequency decomposition coefficient *d* of *c* to filter out the noise; *d* can be calculated according to Formula (1). (**c**) Do wavelet reconstruction using denoised high-frequency decomposition coefficients. The denoised high and low frequency details can be obtained by using Formula (2), and the wavelet reconstruction coefficients can be obtained by Formula (3). Here, H and G generally refer to the low-pass and high-pass filter. $H^{*}$ and $G^{*}$ are the duality operators of *H* and *G*.

At present, there is no uniform standard for the selection of wavelet bases in the processing of well-testing data. The Symlets wavelet function is an approximately symmetric orthogonal wavelet function, which has good local characteristics in the frequency domain. It can reduce phase distortion when analyzing and reconstructing signals [45]. Therefore, we chose the Symlets family for wavelet decomposition and denoising, and the operation flow is shown in Figure 3.

First, under a given range of wavelet disappearance moment M and decomposition scale N of Symlet wavelet, decompose the well-testing data to different scales by using the S. Mallat method and the Birge–Massart soft threshold method. The decomposition formula is as follows:

(1) $$\{\begin{array}{c} C_{\sigma +1,n}=\sum_{k\in Z}h(k-2n)C_{\sigma ,k}\\ D_{\sigma +1,n}=\sum_{k\in Z}g(k-2n)C_{\sigma ,k} \end{array}\begin{array}{c} ,n\in Z \end{array}$$

where $C_{\sigma +1,n}$ is the low-frequency approximation of $C_{\sigma ,k}$, $D_{\sigma +1,n}$ is the high-frequency detail of $C_{\sigma ,k}$, h(k) and g(k) are the low-pass and high-pass filter coefficients under the specified wavelet family, σ is the number of layers of multiscale transformation, $N=2^{\sigma }$, and *n* is the sampling time. Define H=h(k−2n), G=g(k−2n), then Formula (1) can be simplified as:

(2) $$\{\begin{array}{c} C_{\sigma +1}=HC_{\sigma }\\ D_{\sigma +1}=GC_{\sigma } \end{array}$$

Well-testing information is mostly distributed in low-frequency bands, so the low-frequency features contained in the low-frequency wavelet coefficients can better reflect the variation trend of the data segment. The high-frequency features represented by high-frequency coefficients are the noise and interference parts that need to be filtered out by threshold method in the wavelet reconstruction. Polynomials fit the coefficients in the smallest interval containing all non-zero details. Calculate the coefficient matrix of the fitting polynomial and output the high-frequency details $D_{\sigma +1}^{*}$, then the corresponding multiscale reconstruction formula can be simplified as:

(3) $$C_{\sigma }=H^{*}C_{\sigma +1}+G^{*}D_{\sigma +1}^{*}$$

Therefore, after the denoising process, the number of high and low-frequency coefficients would be reduced accordingly, which provides the possibility of data restoration and data compression.

In addition, the vanishing moment *M* is related to the degree of energy concentration and the degree of compression of wavelet coefficients after wavelet transformation. The decomposition scale *N* reflects the overall approximate characteristics and the details of the signal. Here, we used correlation and mean square deviation as indicators, and chose the parameters *M* and *N* with large variance fluctuations (showing that the filtering effect is good) and small average error and mean fluctuations (showing that the reconstructed data are closer to the original data) as the optimal parameters, denoting them as $M_{\mathrm{opt}}$ and $N_{\mathrm{opt}}$.

### 2.2. Kalman Prediction and Smoothing

Kalman is a process of continuous prediction and correction. For each observed pressure variable $x_{j}$, we introduced a corresponding latent variable $z_{j}$. Define node $\left\{z_{j},x_{j}\right\}$ as a variable combination at a specific observation time. Therefore, there always exists a path that connects any two observation variables through latent variables to realize the information interconnection of all oil data. Considering that the latent variables and observed variables follow Gaussian distribution, the joint probability distribution can be obtained as

(4) $$p(x_{1},\cdots ,x_{J},z_{1}\cdots ,z_{J})=p(z_{1})\left[\prod_{j=2}^{J}p(z_{j}|z_{j-1})\right]\prod_{j=1}^{J}p(x_{j}|z_{j})$$

**Figure 4:**
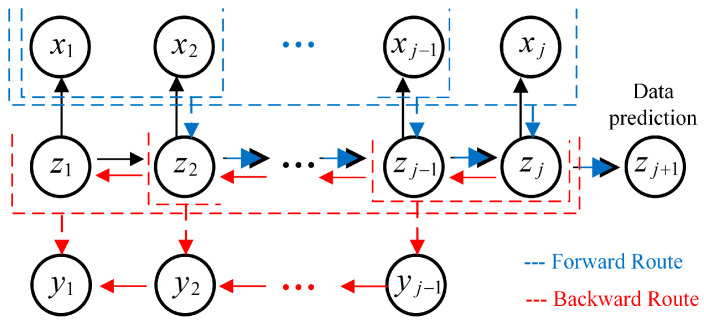
The tree structure interconnecting observed variables and latent variables through forward (**blue line**) and backward **(red line**) routes.

Figure 4 gives the tree structure illustrating the process of Kalman; it includes two processing routes: forward prediction and backward smoothing.

#### 2.2.1. Online Forward Prediction Model

The forward pressure parameters prediction was done in the downhole online test. As shown by the blue path line in Figure 4, the observed variable sequence $x_{1},x_{2}\cdots x_{J}$ was used to derive the variable sequence $z_{1},z_{2}\cdots z_{J}$. Define the transfer information between adjacent observation variables as the normalized marginal probability distribution and denote it as $\hat{\alpha }(z_{j})$. Therefore, what needs to be calculated in the equation $\hat{\alpha }(z_{j})=p(z_{j}|\mu_{j},V_{j})$ is the mean value μ and variance V, and the recursive equation is

(5) $$c_{j}\hat{\alpha }(z_{j})=p(x_{j}|z_{j})\int \hat{\alpha }(z_{j-1})p(z_{j}|z_{j-1})dz_{j-1}$$

where the transition probability is $p(z_{j}|z_{j-1})$, $p(z_{j}|z_{j-1})=N(z_{j}|Az_{j-1},Q)$, the emit probability is $p(x_{j}|z_{j})$, $p(x_{j}|z_{j})=N(x_{j}|Cz_{j},R)$. $c_{j}$ is the normalized coefficient, *A* is the state transition matrix, *C* is the observation transformation matrix, *Q* is the variance of prediction error, *R* is the variance of observation error, $N(x|\mu ,\sigma^{2})$ means that the variable *x* follows the Gaussian distribution with mean μ and variance $\sigma^{2}$. Then, based on $\int p(z_{j}|z_{j-1})\hat{\alpha }(z_{j-1})d_{j-1}=N(z_{j}|A\mu_{j-1},P_{j-1})$, the following relationship can be obtained:

(6) $$\mu_{j}=z_{j}=A\mu_{j-1}+K_{j}(x_{j}-CA\mu_{j-1})$$

(7) $$V_{j}=(I-K_{j}C)P_{j-1}$$

(8) $$P_{j-1}=AV_{j-1}A^{T}+Q$$

(9) $$c_{j}=N(x_{j}|CA\mu_{j-1},CP_{j-1}C^{T}+R)$$

The Kalman prediction gain matrix can be expressed as

(10) $$K_{j}=P_{j-1}C^{T}(CP_{j-1}C^{T}+R)$$

Therefore, for a given $\mu_{j-1}$, $V_{j-1}$, $x_{j}$, the marginal posterior probability of $z_{j}$ could be calculated. With the entry of new observation data, the model would continue to obtain prediction values that reflect the true downhole measuring environment.

#### 2.2.2. Offline Backward Smoothing Model

The backward data smoothing belongs to offline processing and was performed after the pressure parameters are played back to the ground. As shown by the red path line in Figure 4, transfer the information of node $z_{j}$ to node $z_{1}$ in reverse, calculate the Gaussian marginal probability distribution of smooth node $y_{j}$, and record it as $\hat{\beta }(z_{j})$. Then, combine $\hat{\beta }(z_{j})$ and $\hat{\alpha }(z_{j})$ to form an inverse recursive distribution $\gamma (z_{j})$, where,

(11) $$\gamma (z_{j})=\hat{\alpha }(z_{j})\hat{\beta }(z_{j})=N(z_{j}|{\hat{\mu }}_{j},{\hat{V}}_{j})$$

(12) $$U_{j}=V_{j}A^{T}{\left(P_{j}\right)}^{-1}$$

(13) $$c_{j+1}\hat{\beta }(z_{j})=\int \hat{\beta }(z_{j+1})p(x_{j+1}|z_{j+1})p(z_{j+1}|z_{j})dz_{j+1}$$

In the same way, we can obtain

(14) $${\hat{\mu }}_{j}=y_{j}=\mu_{j}+U_{j}({\hat{\mu }}_{j+1}+A\mu_{j})$$

(15) $${\hat{V}}_{j}=V_{j}+U_{j}({\hat{V}}_{j+1}-P_{j})U_{j}^{T}$$

Here, in order to determine the parameters Q and R, we took root mean square error (RMSE) as the fitness index and used PSO to seek for the optimize Kalman parameters. In the PSO algorithm, all particles are given random velocities and positions, and they fly continuously in multi-dimensional space [46]. For the pith particle in the *k*th generation, the PSO algorithm calculates the velocity and position of the *D*th dimension of the *k* + 1th generation according to Formulas (16) and (17).

(16) $$v_{pi,D}^{k+1}=\omega_{p}\times v_{pi,D}^{k}+ac_{1}\times rand()\times (p_{pi,D}^{best}-x_{pi,D}^{k+1})+ac_{2}\times rand()\times (P_{D}^{best}-x_{pi,D}^{k})$$

(17) $$x_{pi,D}^{k+1}=x_{pi,D}^{k}+v_{pi,D}^{k+1}$$

where pi=1,2,3,…,pop; pop is the total number of particles, and $v_{pi,D}^{k}$ and $x_{pi,D}^{k}$ are the velocity and position of the pith particle of the kth generation in the *D*th dimension, respectively. $p_{pi,D}^{best}$ is the best location for pith particles in the *D*th dimension. $P_{D}^{best}$ is the best location for particles in the *D*th dimension. $ac_{1}$ and $ac_{2}$ are acceleration factors. rand() is a random number within the interval [0, 1]. $\omega_{p}$ is the inertia weighting factor. The final optimal position of the population is the optimal search position of the particle swarm.

For the m-th well-testing training sample $X_{m}$, define the fitness value err(m) as shown in Formula (18), where n=1,2…L, $x_{n}^{pred}$ is the Kalman predicted output of $x_{n}$ obtained according to Formula (6). When iterative times are met or the position of the optimal particle that can obtain the minimum value of err(m) is found, and the optimal local Kalman parameters are determined, denote them as $Q_{\mathrm{opt}}^{p}$ and $R_{\mathrm{opt}}^{p}$. Finally, compare all training samples and determine the parameters that can minimize err(m) as the optimal global Kalman parameters. The global optimal solution is given in the “Experimental simulation and results” section.

(18) $$\mathrm{err}\left(m\right)=fitness(X_{m})=(1/L)RMSE(x_{n},x_{n}^{pred})|\mathrm{pop}$$

## 3. Wavelet-Based Kalman Smoothing Measurement System

**Figure 5:**
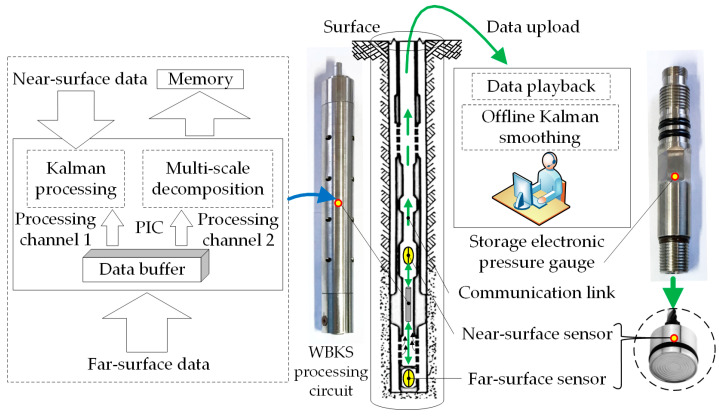
WBKS workflow from data acquisition to data playback based on the well-testing platform.

All samples were collected from the well testing platform of Huabei Oilfield. Each sample included near-surface data and far-surface data, both of which were measured by electronic pressure gauges at the same time but at different well depths. The pressure point number in each sample is from a few score of thousands to hundreds of thousands. Considering that the data obtained from different sensors at different wells or depths have different data sizes *N* and pressure amplitudes *y*, we pre-normalized *N* of each sample at [0, 1000] and *y* at [0, 100]. The data collected by the near-surface sensor are easy to upload but there exists data distortion and redundancy, while the far-surface sensor is closer to the position to be measured and the collected data are more real and effective. Meanwhile, because the cost of pulling out the oil string is large, so in WBKS system, we took the far-surface data as the standard data, and completed the downhole online parameter estimation and the surface offline data smooth processing for near-surface data. The object we multiscale decomposed is the data of one operation stage. The time interval of each stage data is the same and is one second. As shown in Figure 5, the workflow of WBKS signal acquisition and processing can be summarized as:

1. Perform filtering, shaping, and amplification of analog signals collected by Keller sensors.
2. Convert the pressure analog signals into digital signals through an AD converter.
3. Transmit the digital signal to the PIC processor, complete the real-time Kalman forward prediction, and wavelet decompose the pressure signal of one working stage.
4. Store the standard data, the predicted data, and the wavelet coefficients of the data in each working stage into the memory chip.
5. Use the ground software to download all the above stored data and offline Kalman backward smooth the predicted data.
6. Use wavelet coefficients to restore the data, and analyze and compare the data reconstructed by wavelet coefficients with the standard data.

Among them, the storage format of the pressure signal after wavelet decomposition was: “pressure cluster index number + wavelet decomposition coefficient”. The storage location of each pressure cluster was determined by a unique index number. The ground software read the wavelet decomposition coefficients of the corresponding pressure data segment according to the storage format, and sequentially performed data playback, reconstruction, and Kalman smoothing. If a downhole real-time transmission device is equipped to the WBKS measurement system, the new combined system will be able to complete downhole operation decisions and early warnings.

**Figure 6:**
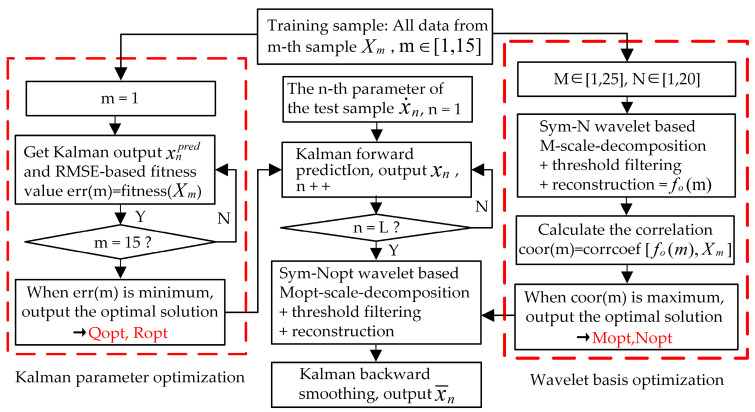
The algorithm flow of WBKS.

This paper used a combination of multiscale analysis and Kalman processing to provide a guarantee for accurately extracting effective information from well test data. The main task was to complete the processing of the collected data. Figure 6 shows the detailed algorithm flow of WBKS. The simulation algorithm of the WBKS model is presented in Algorithm 1.
**Algorithm 1** WBKS algorithm**Pretreatment:** Normalize the data length of all samples to L. **Model Training:** **Input:** m-th well-testing training sample $X_{m}$, m∈[1,15]. **Steps:**  1: Use PSO to obtain $Q_{\mathrm{opt}}^{p}$ and $R_{\mathrm{opt}}^{p}$ under the minimum RMSE fitness err(m).  2: Decompose $X_{m}$ within a given range of decomposition scale M∈[1,25] and vanishing moments N∈[1,20].  3: Get the high-frequency and low-frequency coefficients of the highest decomposition layer, perform Birge–Massart soft threshold processing, and obtain the wavelet reconstruction data $f_{o}(m)$.  4: Calculate the correlation coor(m) between $X_{m}$ and $f_{o}(m)$, where $\mathrm{coor}(m)=\mathrm{corrcoef}(f_{o}(m),X_{m})$.  5: Repeat steps 1–4, select $Q_{\mathrm{opt}}^{p}$ and $R_{\mathrm{opt}}^{p}$ with minimum err(m) as the optimal Kalman parameter, denote them as $Q_{\mathrm{opt}}$ and $R_{\mathrm{opt}}$; select *M* and *N* with maximum coor(m) as the optimal combination of wavelet reconstruction parameters, denote them as $M_{\mathrm{opt}}$ and $N_{\mathrm{opt}}$. **Model Testing:**
**Input:** The n-th data point ${\dot{x}}_{n}$ of testing sample $\dot{X}$. **Steps:**  1: Use $Q_{\mathrm{opt}}$ and $R_{\mathrm{opt}}$ to calculate the predicted value $x_{n}$ of ${\dot{x}}_{n}$ and restore it.  2: If *n* = L, do $\text{sym-}N_{\mathrm{opt}}$ wavelet-based $M_{\mathrm{opt}}$-scale-decomposition, soft threshold filtering, and reconstruction on $\dot{X}$, save the reconstructed wavelet coefficients.  3: Use Formula (14) to smooth $\dot{X}$ and obtain ${\bar{x}}_{n}$.

## 4. Experimental Simulations and Results

This article analyzed 15 field samples obtained on the spot. All data training and test results were obtained by MATLAB 2017a simulating.

**Figure 7:**
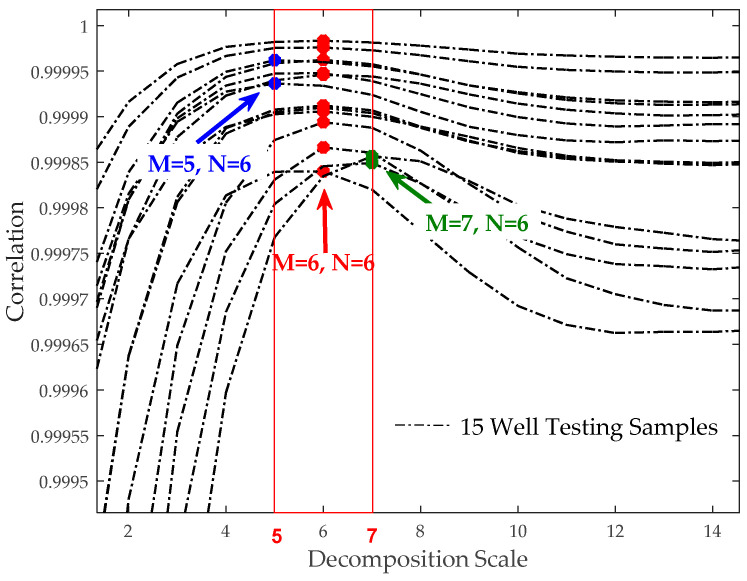
Correlation comparison of different decomposition scales using a sym6 wavelet basis.

Figure 7 shows the distribution of the best decomposition scale when N = 6 and 15 oil well-testing samples obtaining the maximum correlation. When the decomposition scale was from 5 to 7, the correlation value was the highest, showing that the reconstruction coefficients obtained under these decomposition scales have a good ability to restore data.

**Figure 8:**
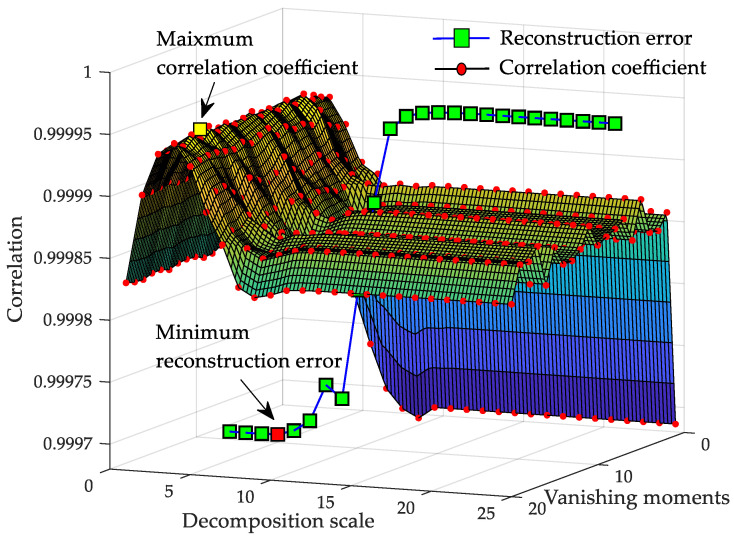
Correlation coefficient and reconstruction error trend under Symlets wavelet processing.

Figure 8 shows the symlets wavelet-based correlation coefficients distribution at different decomposition scales and vanishing moments, M∈[1,25], N∈[1,20]. We randomly selected one of the samples for correlation analysis. Before processing, the pressure range of the sample is normalized to [0,1]. It can be seen that the distribution of correlation coefficient presented a “bed shape”: with the increased of the vanishing moment order, the energy of the wavelet reconstruction signal was more concentrated and finally, tended to be stable. A too large decomposition scale would not only reduce the processing efficiency, but also filter out more small but important wavelet coefficients details, resulting in the loss of important original information and the decline of the correlation between the signals before and after reconstruction.

Figure 8 also shows the mean square error trend of reconstructed data based on a sym6 wavelet basis. When the decomposition scale is small, due to incomplete decomposition, a large number of original characteristics are still retained in the reconstruction coefficients, so the reconstruction root mean square error was not fluctuated significantly and the noise reduction effect was not obvious. The experimental results are consistent with the analysis of the decomposition principle, which proves that it is feasible to choose correlation as a wavelet denoising standard in this paper.

To determine the optimal decomposition scale, we took the average error ${\bar{e}}_{i}$, the average value fluctuation rate $\phi_{i}^{mean}$, and the variance value fluctuation rate $\phi_{i}^{std}$ as indicators, reconstructed all the near-surface samples and analyzed the ability of the reconstructed samples to represent the original samples. Their formulas are defined as follows:

(19) $${\bar{e}}_{i}=\sum_{j=1}^{L_{i}}abs[f_{0}(i,j)-X(i,j)]/L_{i}$$

(20) $$\phi_{i}^{mean}=abs[\varphi_{mean}^{ori}(i)-\varphi_{mean}^{rec}(i)]/\varphi_{mean}^{ori}(i)$$

(21) $$\phi_{i}^{std}=abs[\varphi_{std}^{ori}(i)-\varphi_{std}^{rec}(i)]/\varphi_{std}^{ori}(i)$$

where *i* is the serial number of the well-testing sample i∈[1,15], *j* is the index number of the data point in each sample, $\varphi_{mean}^{ori}(i)$ is the mean of the original sample, $\varphi_{mean}^{rec}(i)$ is the mean of the reconstructed sample, $\varphi_{std}^{ori}(i)$ is the variance of the original sample, and $\varphi_{std}^{rec}(i)$ is the variance of the reconstructed sample. Table 1 shows the comparison effects of 15 well-testing samples before and after the wavelet reconstruction. The samples with both M and N configured as 6 were in majority and had achieved good results in reducing the average error of reconstruction and avoiding too much fluctuation in the mean and variance. Therefore, the optimal decomposition scale $M_{\mathrm{opt}}$ and the optimal vanishing moment $N_{\mathrm{opt}}$ were both determined to be 6.

We took the 5-th order polynomial fitting and total 20 fitting points as an example. Firstly, the $M_{\mathrm{opt}}$ and $N_{\mathrm{opt}}$ were used to perform wavelet decomposition and wavelet coefficient fitting on the fourth sample in Table 1 containing 33,996 pressure points. Then, the fitted coefficients were written in PIC microcontroller as the configuration parameters. In this way, 25 parameters represented the main information of original signal with 33,996 points. Only 25 parameters need to be recorded in memory, and the compression ratio is about 136:1. Formula (22) gives the formula for calculating the value *y* at *x*, where *n* represents the order. The values of the 3-th and 5-th order polynomial fitting coefficients are respectively given in Formulas (23) and (24).

(22) $$y=p1*x^{n}+p2*x^{\left(n-1\right)}+\ldots +pn*x+p(n+1)$$

(23) $$\mathrm{when}\;n=3,\;\{\begin{array}{c} p1=-4.084238503305881*10^{-5},p2=0.110554372435346,\\ p3=-84.889040506617850,\;p4=1.048853034985732*10^{5}. \end{array}$$

(24) $$\mathrm{when}\;n=5,\;\{\begin{array}{c} p1=1.220929921783823*10^{-10},p2=-9.684472568414146*10^{-7},\\ p3=0.002549151637694,\;p4=2.738185240847735,\\ p5=1.099908486472324*10^{3},p6=-1.113382621476452*10^{4}. \end{array}$$

**Figure 9:**
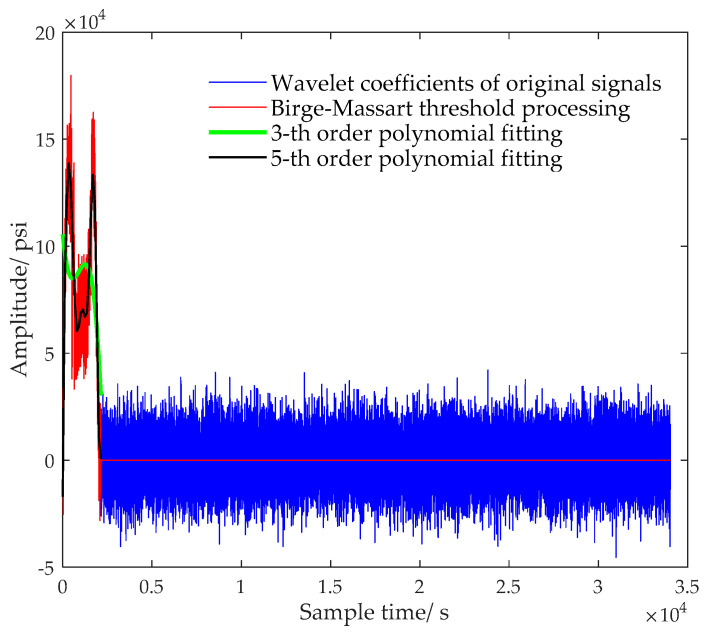
The distribution of wavelet coefficients under different reconstruction methods.

Figure 9 reflects the distribution of wavelet coefficients when different reconstruction methods were used. In it, the number of the obtained coefficients varied with different methods; there were 34,093 original wavelet coefficients, 2135 threshold coefficients after forced denoising, and 20 coefficients after the 3-th or the 5-th order polynomial fitting and quadratic wavelet reconstruction. The 5-th order polynomial could well characterize the trend of wavelet characteristics, while the data reconstructed by the 3-th order polynomial had a large deviation from the original data. Here, being an example, the 5-th order polynomial fitting was put forward just for illustrating the feasibility of fast data compression. The optimal order of the polynomial was not determined and should be flexibly adjusted according to the memory size and production accuracy requirements.

**Figure 10:**
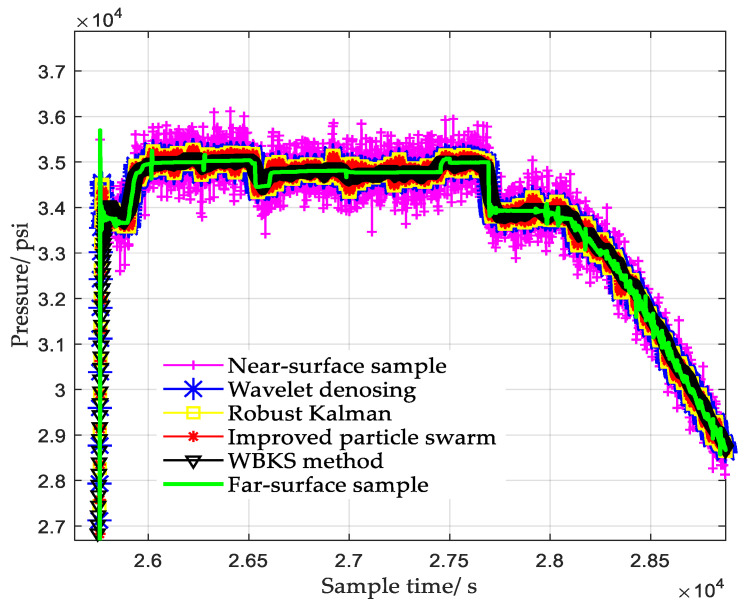
Output comparison under different processing methods.

**Figure 11:**
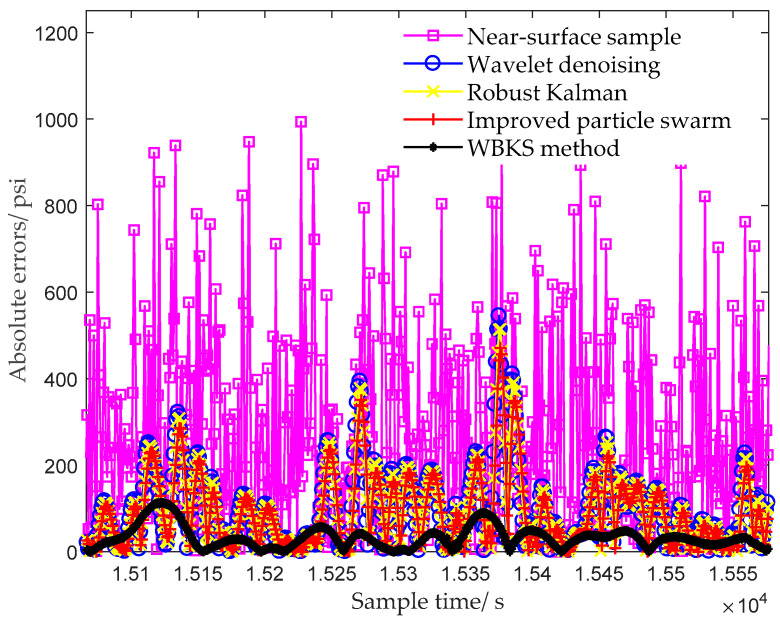
Absolute errors comparison under different processing methods.

Figure 10 and Figure 11 took the far-surface data as the standard data, compared the filtering effect and absolute error on the near-surface data of the fourth sample after using different processing methods. The wavelet denoising method sequentially performed sym6-based 6-scale wavelet decomposition, coefficient soft threshold processing, and coefficient reconstruction. The classical robust Kalman method used Formula (6) to calculate the filter outputs, and its Q and R were random values. The improved particle swarm algorithm, on the basis of the classical Kalman method, added the optimization of Q and R by PSO. It can be seen from Figure 10 that due to random interference and mechanical vibration, the near-surface sample contained a lot of burrs before being processed by other methods, resulting in the data having poor resolution and high absolute error. From the processing results of near-surface data, we know that the filtering capabilities and the abilities to restore the true pressure trend of different methods were different. Among all the processed data, the data processed by the WBKS method were closest to the real far-surface data, and its absolute error was also the smallest.

**Figure 12:**
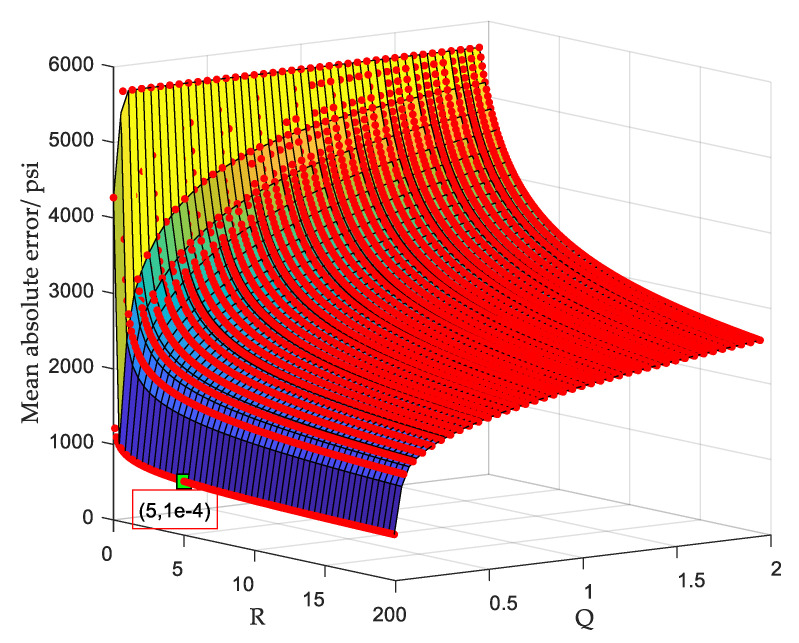
Distribution of mean absolute error under traversing Q and R.

In order to intuitively show the position of the optimal parameters obtained using PSO in all parameters, we took the same fourth sample as the analysis object and used the traversal method to plot the average absolute error distribution under different Q and R values. R∈[0,20] and Q∈[0,2], the traversal steps of Q and R, were 0.05 and 0.1, respectively. The experimental results in Figure 12 show that the obtained optimal parameters were $Q_{\mathrm{opt}}=10^{-4}$ and $R_{\mathrm{opt}}=5$.

To test the robustness of the WBKS method, the fourth near-surface sample $x_{i}$ (*i* = 4), added with different noise intensity, were used as the new signals to be analyzed. As shown in Table 2, we took error and signal-to-noise ratio as the analysis indicators, and used various methods to process new signals and compared their results. The definitions of various analysis indicators are shown in Formulas (25)–(27), which mainly include Mean Absolute Deviation (MAD), Standard Deviation (SD), and Signal-to-Noise Ratio (SNR). $x_{i,j}^{o}$ is the *j*-th pressure value from the *i*-th far-surface sample. ${\bar{x}}_{i}^{o}$ is the mean value of all pressure values from the *i*-th far-surface sample, and $x_{i,j}^{n,\tau }$ is the *j*-th pressure value after adding noise with intensity coefficient τ to the *i*-th near-surface sample. The noise intensity coefficient represents the maximum amplitude ratio of random noise to near-surface data. Opt/Exp indicates the analysis indicators values obtained by different methods configured with the global or local optimal parameters. Optimal (Q, R) represents the local optimal Q and R values suitable for the Kalman model processing $x_{i,j}^{n,\tau }$, which is obtained by using PSO.

(25) MAD_τ=1Li∑j=1Liabs(xi,jo−xi,jn,τ)

(26) SD_τ=[1Li∑j=1Li(xi,jo−xi,jn,τ)2]1/2

(27) $$SNR\_\tau =10\log_{10}\left(\sum_{j=1}^{L_{i}}{\left(x_{i,j}^{o}-{\bar{x}}_{i}^{o}\right)}^{2}/\sum_{j=1}^{L_{i}}{\left(x_{i,j}^{o}-x_{i,j}^{n,\tau }\right)}^{2}\right)$$

## 5. Discussion

To achieve the requirements of oil data compression and real-time upload, we built a well-testing measurement system and presented a wavelet-based Kalman data smoothing method. With the practical application of WBKS in the oil well-testing operation platform, its model construction, system parameter optimization, various methods comparison, and system performance analysis have been performed in this paper.

Firstly, multiscale analysis and wavelet transform theory were discussed. For data denoising, utilizing coefficients under wavelet transform made the WBKS have a good noise-reducing ability and the complete trend maintenance characteristic. Thresholding the extracted wavelet coefficients was both forthright and reliable and could be easily transplanted to the PIC unit. For data compression, the parameter fitting case in Figure 9 shows that the threshold processing makes the amount of data that needs to be uploaded or stored reduced from 33,996 to 25, which indirectly expands the storage capacity and can upload the data to the computer through the serial port for further processing. Meanwhile, to improve working curve resolution, the influence of using different wavelet scales and vanishing moments on denoising the well-testing data were analyzed. Fifteen near-surface samples were used as testing sets, based on which, the results in Figure 7 demonstrated that, the best combination of parameters M and N had a narrow value range, M∈[5,6], N∈[6,7]. These determined ranges can provide technical reference for wavelet decomposition of uncertain downhole data. For data storage, the memory circuit saved the original data, Kalman prediction data, fitting parameters of wavelet decomposition coefficients, which was convenient for further analysis, statistics, and storage of data. This storage form provides a long-term effective data basis for algorithm verification and parameter optimization.

In Kalman parameters optimization, the improved particle swarm method used PSO to optimize Q and R of the Kalman model. After that, based on indicator RMSE and the criterion of minimizing the fitness values, the best Kalman parameters were determined. Compared to classical Kalman method and wavelet denoising method, although the improved particle swarm method reduced the deviation from the original data (see results in Figure 10 and Figure 11) and the configured Kalman filter parameters were locally optimal, there was still a big deviation from the original signal. The wavelet denoising and Kalman smoothing methods used in WBKS complement each other, which not only remove the noise but also retain the valid data information. Therefore, WBKS could purify and highlight the inherent characteristic information of the signal. After WBKS processing, the output absolute error was smaller, and the signal curve was more “stable” and closer to the real well-testing data.

From the processing results of a single algorithm, Kalman performed more effectively in eliminating noise and its performance was better than that of wavelets. After wavelet processing, although most of the noises in the original data had been eliminated, the error comparison results showed that its noise filtering was not thorough. It may be that the frequency of the noise covered almost the entire frequency axis and was not easily separated. The multi-sample training and the global optimal parameters selection were adopted after the PSO based local optimal parameters seeking, this ensured that the method could improve system efficiency and lead the system to achieve optimal performance. Therefore, the Kalman smoothing method using the global optimal parameters had better analysis and denoising capabilities than using the local optimal parameters, and obtained better processing results (see Table 2).

In the WBKS model, the wavelet first prefiltered out most of the high-frequency noise and system deviations of the near-surface data, which laid the foundation for the next Kalman operation. Table 2 shows that adding the Kalman filter to further process the wavelet reconstructed data made the WBKS model more accurately forecast the dynamic changes of oil parameters while retaining valid data information. Figure 10 intuitively shows that the processing data curve which was calculated from the WBKS method was the most consistent with the far-surface data curve and the method well-simulated the pressure change rule of the downhole measurement system. The smallest absolute error in Figure 11 corroborates the effectiveness of the WBKS method. In Table 2, when τ=0.05, compared with the near-surface data, the absolute error of the data processed by wavelet was reduced by 74.6%, the reduction of Kalman forward prediction was 75.4%, and that of Kalman backward smoothing was 85.2%. Similarly, when τ=0.3, the reductions of the above three comparisons were 75.6%, 77.1%, and 86.5%. The obtained high data characterization capability and low data restore error of WKBS are benefits from the combination of wavelet and Kalman.

In order to evaluate the results of pretreatment on a single sample and obtain the applicability and performance conclusion of each method, one far-surface sample was used as a testing set, and in the comparative analysis of different methods, three definitions including Mean Absolute Deviation, Standard Deviation, and Signal-to-Noise Ratio were given. For different methods with the same noise intensity coefficient, the low MAD and SD and high SNR in Table 2, respectively, reflect the proposed method’s strong data restoration ability and denoising ability. During Kalman processing, the forward recurrence used only part of the observation data to complete the data prediction, while the reverse recursion was to complete the data smoothing on the basis of obtaining all the observation data. Therefore, in the case of using the same configuration parameters, the method that added backward smoothing had better results than that of using the forward prediction alone. For one method with different noise intensity coefficients, the WBKS method had good performance under all evaluation indexes and had the characteristics of strong data restore ability, high robustness, and large signal-to-noise ratio, which proved the feasibility of its application in oilfield development.

The proposed WBKS method is suitable for applications in signal processing and communication where simultaneous estimation, decomposition, and compression are desired. However, there are inevitably some limitations, mainly reflected in three aspects:

1. The complex operational processes.

The played-back data in this paper were obtained by manual download after the downhole measurement was completed and the pressure gauge was pulled out to the surface. In terms of the measurement system, frequently lowering and lifting the pressure gauge will increase the operating burden of the workers and may cause uncertain events such as pipe-stuck and lost circulation.

2. The necessity of further research on data compression.

The main works of this paper are data denoising, prediction, and smoothing. The method of compressing data was not described in detail, but only proved the feasibility of using polynomial fitting to complete data compression.

3. The samples used for the study are few.

In actual production, due to obtaining a complete downhole well-testing data being difficult and requiring a long acquisition cycle, there are only 15 analyzable samples in this paper.

Therefore, in the future, three aspects of work corresponding to the above limitations need to be further done. First, to realize the data real-time upload with zero human intervention, we are planning to introduce transmission systems such as LWD or some wireless transmission modules to reduce project cost and production accidents. Second, we are going to analyze the deviation error between the original data and the restored data obtained using different compression methods with different compression ratios. It is very practical to use compression theory in accordance with actual production requirements to ensure data compression quality. Third, we should increase the number of samples to make the obtained processing model and optimization parameters more convincing.

## 6. Conclusions

To ensure that the processing results of well-testing data are in line with the dynamic changes of actual downhole geological parameters, we first determined the optimal combination with the mother wavelet (sym6) and the wavelet decomposition scale of 6 by analyzing the data reconstruction effects (involving correlation and reconstruction errors MAD, SD, and SNR) under different decomposition scales and disappearing moments. Secondly, we optimized the parameters of the Kalman prediction and smoothing model using PSO, and obtained the optimal parameters Q and R suitable for processing the well-testing data. Then, we configured all the obtained optimal parameters to the pressure gauge and completed the downhole online wavelet decomposition, fitting, reconstruction, Kalman prediction, and data storage. After the above downhole operations, the playback data were smoothed offline on the surface. The experimental results show that wavelet decomposition and reconstruction using optimal parameters can achieve high-efficiency data compression and low-error data restoration, meeting the requirements of real-time transmission and accurate playback. The optimized Kalman provided effective technical support for removing noise, improving data resolution, enhancing robustness, and completing accurate data interpretation. The proposed WBKS not only opens up new ideas for data compression and real-time data transmission in well-testing operations through multiscale decomposition and reconstruction methods, but also provides important guidance for decision-making and early warning of petroleum working conditions based on Kalman estimates.

## Figures and Tables

**Table 1:** The comparison of the wavelet reconstruction effects under different M and N combination.

No./*i*	M	N	${\bar{e}}_{i}$	$\varphi_{mean}^{ori}(i)$	$\varphi_{std}^{ori}(i)$	$\varphi_{mean}^{rec}(i)$	$\varphi_{std}^{rec}(i)$	$\phi_{i}^{mean}$	$\phi_{i}^{std}$
1	5	6	0.007869	0.743473	0.260655	0.736068	0.247624	0.00996	0.049996
2	5	6	0.020657	0.355812	0.285103	0.376231	0.273351	0.057387	0.041222
3	6	6	0.016471	0.554035	0.276538	0.546707	0.256993	0.013227	0.070677
4	6	6	0.007137	0.614972	0.189785	0.614416	0.179372	0.000904	0.054869
5	6	6	0.019565	0.445155	0.394536	0.464322	0.378328	0.043057	0.04108
6	6	6	0.016843	0.361023	0.120409	0.377780	0.115105	0.046416	0.044053
7	6	6	0.012392	0.387651	0.135597	0.399926	0.128853	0.031665	0.049736
8	6	6	0.013925	0.573745	0.236822	0.580321	0.224591	0.011462	0.051645
9	6	6	0.009512	0.444279	0.159682	0.452616	0.151253	0.018765	0.05279
10	6	6	0.011399	0.598858	0.253192	0.606597	0.241222	0.012923	0.047277
11	6	6	0.012743	0.615074	0.189796	0.607654	0.178122	0.012065	0.061505
12	6	6	0.011399	0.598858	0.253192	0.606597	0.241222	0.012923	0.047277
13	6	6	0.023324	0.673282	0.342663	0.663130	0.320238	0.015079	0.065445
14	7	6	0.015583	0.355828	0.091037	0.371376	0.086595	0.043696	0.048802
15	7	6	0.011324	0.420021	0.107204	0.431263	0.101705	0.026765	0.051297

**Table 2:** Robust analysis of the WBKS algorithm based on multiple analysis indicators.

Indicators\Results	Local Optimal (Q,R)	Near-Surface Sample	Robust Kalman	Improved Particle Swarm	Wavelet Denoising	Forward Prediction (Opt/Exp)	Backward Smoothing (Opt/Exp)
MAD_0.05	(0.0134,2.0081)	1420.701	949.979	311.85	361.432	349.857/391.832	210.616/347.559
SD_0.05		1783.52	1192.869	517.568	480.205	524.804/513.565	381.788/463.783
SNR_0.05		14.507	18.001	25.251	25.901	25.13/25.318	27.891/26.203
MAD_0.1	(0.00534,1.996)	2831.115	1887.271	485.871	706.201	667.772/786.888	395.003/650.600
SD_0.1		3550.103	2368.319	753.082	905.461	894.251/1003.713	561.863/835.376
SNR_0.1		8.528	12.044	21.996	20.396	20.504/19.501	24.54/21.095
MAD_0.15	(0.0024,2.9664)	4276.761	2858.103	581.319	1049.631	1003.506/1180.530	590.073/891.840
SD_0.15		5351.622	3575.044	919.306	1333	1279.415/1495.191	783.751/1132.575
SNR_0.15		4.964	8.468	20.264	17.037	17.393/16.039	21.649/18.452
MAD_0.2	(0.0029,5.9815)	5720.959	3851.621	685.573	1437.723	1351.764/1611.532	779.812/1144.366
SD_0.2		7163.155	4813.474	1123.106	1818.597	1715.289/2032.833	1006.072/1439.780
SNR_0.2		2.431	5.884	18.525	14.339	14.864/13.371	19.481/16.367
MAD_0.25	(0.0102,4.5979)	7131.965	4758.136	749.637	1784.697	1671.759/1968.838	962.176/1314.854
SD_0.25		8932.155	5964.693	1225.635	2251.017	2106.425/2480.451	1224.626/1658.106
SNR_0.25		0.514	4.021	17.761	12.484	13.061/11.641	17.768/15.138
MAD_0.3	(0.00137,2.0119)	8525.206	5694.416	1033.786	2084.213	1958.073/2348.266	1148.449/1722.288
SD_0.3		10,700.338	7144.135	1407.038	2620.212	2448.163/2946.728	1439.265/2148.962
SNR_0.3		−1.055	2.454	16.567	11.166	11.756/10.146	16.37/12.888
